# Efficacy and safety of intravenous sufentanil administration in low-severity acute trauma as a competence of paramedics: a follow-up observational study

**DOI:** 10.1007/s00068-025-02953-0

**Published:** 2025-09-09

**Authors:** Roman Sykora, Nikola Kukackova, Ondrej Sopko, David Peran, Jiri Smetana, Metodej Renza, Milos Kukacka

**Affiliations:** 1 Department of Anesthesia and Intensive Care, Third Faculty of Medicine, Charles University and FNKV University Hospital, Prague, Czech Republic; 2 Emergency Medical Services of Karlovy Vary Region, Zavodni 390/98C, Karlovy Vary, 36006 Czech Republic; 3 Medical College, Prague, Czech Republic

**Keywords:** Paramedic competencies, Pre-hospital analgesia, Opioid, Sufentanil, Low-severity trauma

## Abstract

**Background and importance:**

In the Czech Republic, paramedics are required to consult a physician before administering intravenous opioids, which may delay effective prehospital pain management. As paramedic competencies expand in Europe, it is important to evaluate the safety and efficacy of independent opioid administration in prehospital emergency care settings.

**Objectives:**

To assess the safety and effectiveness of intravenous sufentanil administered independently by trained paramedics compared to administration following remote physician consultation in adult trauma patients.

**Design:**

Prospective, single-center, observational cohort study.

**Settings and participants:**

Conducted at the Emergency Medical Services of the Karlovy Vary Region between January 1 and December 31, 2024. The study included 462 adult trauma patients who were hemodynamically stable and conscious. Patients were divided into two groups: the Consultation group (physician consultation required) and the Competency group (paramedics administering independently).

**Intervention or exposure:**

Intravenous administration of sufentanil, with or without physician phone consultation.

**Outcome measures and analysis:**

Primary outcomes included reduction in pain (measured using the Numeric Rating Scale, NRS) and incidence of adverse events (e.g., respiratory depression, oxygen desaturation, hypotension, and antiemetic use). Statistical significance was set at *p* < 0.05.

**Main results:**

Both groups achieved similar pain reduction. The Competency group received a higher mean dose of sufentanil (9.7 ± 3.0 µg vs. 8.9 ± 2.8 µg; *p* = 0.006) and more frequently used non-opioid adjuvant analgesics (54% vs. 41%). Documentation of pain scores was significantly better in the Competency group (87% vs. 43%; *p* < 0.01). Adverse events were rare, non-serious, and comparable between groups. Antiemetics were more frequently administered in the Competency group (11% vs. 6%; *p* = 0.037). A minor, clinically insignificant reduction in diastolic blood pressure was observed in the Competency group.

**Conclusion:**

Intravenous sufentanil administered independently by trained paramedics in adult patients with lower severity trauma demonstrated safety and effectiveness comparable to administration following remote physician consultation, along with improved documentation and increased use of multimodal analgesia. Although this was a monocentric study, these observations may contribute to ongoing discussions about expanding paramedic competencies in opioid analgesia within the Czech prehospital emergency system.

**Supplementary Information:**

The online version contains supplementary material available at 10.1007/s00068-025-02953-0.

## Introduction

In the Czech Republic, current legislation does not permit paramedics operating without a physician to administer intravenous opioids in prehospital emergency care (PEC). This limitation presents a significant challenge in the management of acute traumatic pain, as paramedics must consult a remote physician by phone before initiating opioid analgesia. In contrast, several European countries allow paramedics to administer opioid analgesics independently, with authorization typically based on specific training and protocol-driven approaches [1, 2]. 

Previous pilot studies in the Czech Republic, as well as research conducted by German team [3], have explored this issue [4]. Notably, recent changes in German legislation have enabled paramedics to administer opioid analgesia in defined situations without direct physician oversight [5]. Given the structural and procedural similarities between the Czech and German PEC systems, this remains a timely and relevant topic in the Czech context.

In the Czech Republic, as in many other European countries, sufentanil is among the most commonly used opioids for managing traumatic pain in the PEC setting [6, 7]. This study seeks to contribute to the growing body of evidence informing the potential extension of paramedic competencies within the Czech system in European context [8]. Specifically, it investigates the clinical effectiveness and safety of autonomous intravenous administration of sufentanil by appropriately trained paramedics in adult patients presenting with moderate acute traumatic pain, following a standardized protocol.

## Methods

Since 2020, Emergency Medical Services (EMS) of the Karlovy Vary Region in the Czech Republic has been piloting the use of opioid treatment for pain management in trauma cases [4]. This competency is optional for paramedics and is granted following a structured training process [4]. The process includes e-learning modules, in-house lectures, and simulation-based sessions, all of which involve assessment of both knowledge and practical skills. Re-certification was required for paramedics every twenty-four months. Participation data such as age, gender, years of experience, and level of medical education (university degree or higher professional qualification) were tracked among paramedics.

### Study design

This study was conducted in accordance with the STROBE guidelines to ensure transparent and complete reporting [9]. This study was designed as a single-center, prospective, observational cohort trial with two parallel groups. The study protocol and its implementation were approved by the Ethics Committee of the EMS of the Karlovy Vary Region, registered with the State Institute for Drug Control of the Czech Republic, on August 11, 2024 (Reference No. ZZSKVK/EK/01/2024). In accordance with Czech legislation (Health Services Act No. 2011, 372 (CZ), informed consent was not required from patients presenting with acute traumatic pain in PEC [10]. Instead, participation was based on the principle of tacit consent, following the provision of standard information about the proposed procedures. The trial was registered at ClinicalTrials.gov (Identifier: NCT06514469) on July 16, 2024.

### Study location

The study was conducted at the EMS of the Karlovy Vary Region, Czech Republic, over a 12-month period from January 1, 2024, to December 31, 2024.

### Data source and eligibility criteria

All data were retrieved from the electronic patient documentation system *ePaRe* (part of MZD, European Medical Distribution Ltd., Bratislava, Slovak Republic). Patients were eligible for inclusion if they met the following criteria: (a) sufentanil was administered by paramedics on scene without the physical presence of a physician, (b) the patient was at least 18 years old, (c) the patient was fully conscious (defined as Alert with a Glasgow Coma Scale score of 15), and (d) the patient was hemodynamically stable (systolic blood pressure > 100 mmHg and no bradycardia, i.e., heart rate ≥ 60 bpm). Consequently, two study groups were identified based on the method of sufentanil administration: *Consultation* group – patients who received sufentanil administered by paramedics after telephone consultation with an EMS physician, and *Competency* group – patients who received sufentanil administered independently by trained and authorized paramedics.

In the *Competency* group, paramedics were allowed to administer intravenous sufentanil in titrated doses up to a maximum of 20 micrograms. Administration was considered appropriate when the patient’s reported pain score on the Numeric Rating Scale (NRS) was greater than 4 points.

Baseline characteristics were collected from electronic patient records, including age, sex, NACA score (National Advisory Committee for Aeronautics), type of injury (upper or lower limb, torso, head or burns), sufentanil dose and whether it was administered fractionally, and the proportion of cases in which additional non-opioid analgesia (paracetamol) was used.

### Outcome measures

Ambulance response times and on scene times, the sufentanil dosing and additional analgesia were extracted. Efficacy was evaluated by the frequency of complete Numeric Rating Scale (NRS) pain assessments, recorded both prior to sufentanil administration and at patient handover. The safety outcomes included the incidence of respiratory arrest (defined as the need for bag-valve-mask ventilation), bradypnea (respiratory rate < 10 breaths per minute), and the requirement for oxygen therapy (SpO₂ <92%). Recorded adverse effects were assessed, including the incidence of nausea and vomiting and the need for intravenous antiemetic treatment. Furthermore, changes in physiological parameters, including heart rate, blood pressure, peripheral oxygen saturation (SpO₂), and respiratory rate, were recorded from initial assessment through to patient handover.

### Data presentation and statistical analysis

Continuous variables are presented as mean ± standard deviation, while categorical variables are expressed as counts (n) and percentages. The Kolmogorov–Smirnov test was used to assess the normality of continuous data. Depending on the data type and distribution, appropriate statistical tests were applied, including the t-test, Chi-square test, and Fisher’s exact test. A p-value of < 0.05 was considered statistically significant. All analyses were conducted using STATISTICA 7.0 (StatSoft, Inc., Tulsa, Oklahoma, USA). The sample size was not calculated in advance; however, the protocol initially defined a target of 100 cases per group and a data collection period of six months. Subsequently, following agreement within the research team, the study period was extended to one year.

## Results

**Fig. 1:**
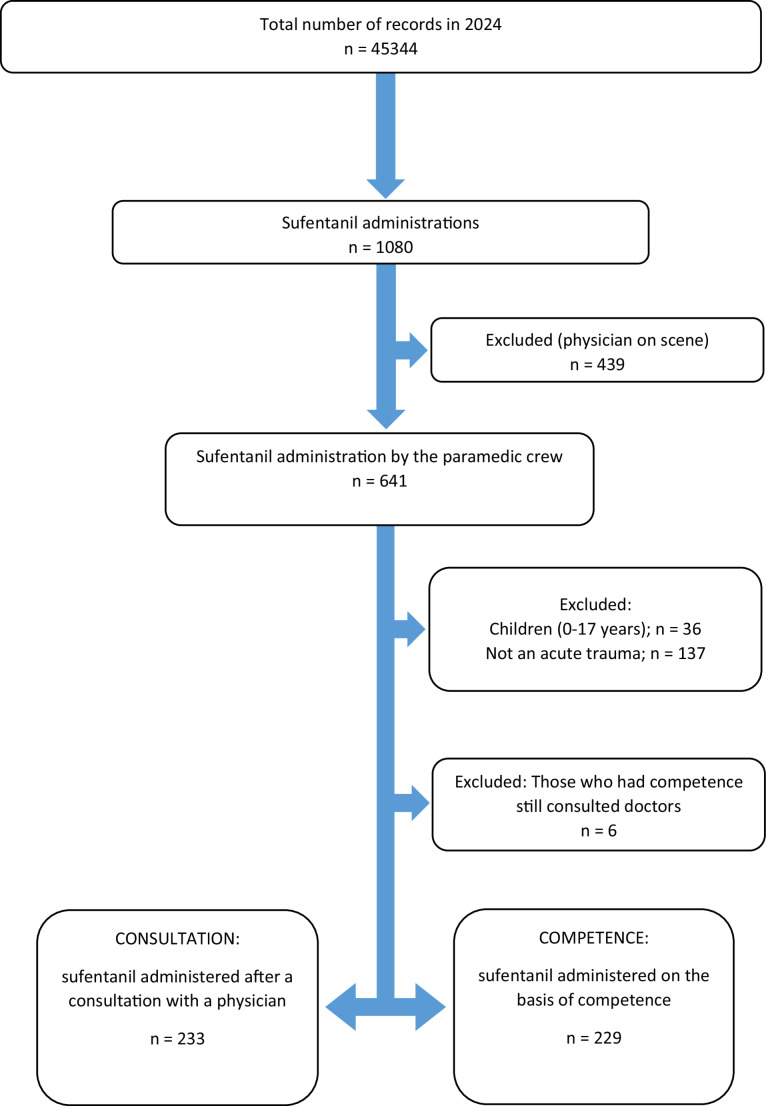
Identification of patient groups with acute trauma receiving sufentanil administered exclusively by paramedics without on-scene physician involvement

In the observed year (2024), 112 paramedics worked in the ambulance crew, with half having the competency to administer sufentanil independently (*n* = 56). Details and a comparison of paramedics with and without competency are shown in Table S1 of the supplementary material. The selection of study groups is outlined in the study flow diagram (Fig. 1). Of the total 45,344 EMS interventions in 2024, 233 cases of sufentanil administration occurred in the *‘Consultation’* group (where a physician was consulted by phone), and 229 cases in the *‘Competence’* group (administered by paramedics with competency). The baseline characteristics of both study groups are summarized in Table 1.

**Table 1:** Baseline characteristics

	Consultation (*n* = 233)	Competence (*n* = 229)	*p*-value
Age (years)	62.9 ± 21.4	64.1 ± 20.0	NS
Sex (women)	136 (58%)	143 (62%)	NS
Ambulance response time (min)	10.5 ± 5.3	10.6 ± 5.1	NS
NACA	2.9 ± 0.5	2.8 ± 0.4	NS
Trauma of lower extremity	158 (68%)	145 (63%)	NS
Trauma of upper extremity	45 (19%)	53 (23%)	NS
Trauma of torso	21 (9%)	26 (11%)	NS
Trauma of head	5 (2%)	5 (2%)	NS
Burns	4 (2%)	--	NS

Data are presented as mean ± standard deviation or as number (percentage)

*Abbreviations*: *MDC* Medical Dispatching Center; *NACA* National Advisory Committee for Aeronautics score; *NS* not significant

Despite the mandatory telephone consultation, paramedics in the *Consultation* group spent a comparable amount of time on scene as those authorized to administer sufentanil independently (*Competency*). However, the dose of intravenously administered sufentanil in the *Consultation* group was significantly lower than in the independent competence group (8.9 ± 2.8 µg vs. 9.7 ± 3.0 µg, *p* = 0.006). Non-opioid adjuvant analgesia was less frequently administered in the *Consultation* group (41% vs. 54%). Although the reduction in NRS scores was calculated to be similar in both groups, complete NRS documentation was significantly less frequent in the *Consultation* group (43% vs. 87%, *p* < 0.01) (Table 2).

**Table 2:** On-scene time, characteristics of analgesia, and pain reduction

	Consultation (*n* = 233)	Competence (*n* = 229)	*p*-value
Ambulance on scene time (min)	26.0 ± 9.8	25.1 ± 9.5	NS
Dose of sufentanil (µg)	8.9 ± 2.8	9.7 ± 3.0 *#*	0.006
Fractional administration (documented)	65 (28%)	74 (32%)	NS
Additional i.v. paracetamol use	79 (34%)	124 (54%) *#*	< 0.001
Other additional analgesics	17 (7%)	0 *#*	< 0.05
NRS, initial (points 0–10)	7.6 ± 1.7	7.4 ± 1.8	NS
NRS reduction (points)	−3.8 ± 1.9	−4.0 ± 1.8	NS
Complete NRS reporting	100 (43%)	199 (87%) *#*	< 0.001

Data are presented as mean ± standard deviation or as number (percentage)

*Abbreviations*: *NRS* Numeric Rating Scale for subjective pain assessment; *i.v.* intravenous; *NS* not significant

*# *Statistically significant with presented p-value

Adverse events such as respiratory depression, need for oxygen therapy, hypotension, nausea, or vomiting were infrequent and occurred at similar rates in both groups. However, intravenous antiemetics were administered more often in the *Competence* group (11% vs. 6%; *p* = 0.037) (Table 3). A reduction in diastolic blood pressure prior to handover was observed in the *Competence* group (−2.5 ± 9.6 vs. + 1.8 ± 8.2 mmHg), while other vital signs including, systolic blood pressure, heart rate, respiratory rate, SpO₂, and GCS, remained unchanged (Table 4 and Supplementary material Table S3).

**Table 3:** Adverse events and their treatment following intravenous sufentanil use in trauma care

	Consultation (*n* = 233)	Competence (*n* = 229)	*p*-value
Respiratory arrest	-	-	-
Bradypnea, hypoventilation	-	1 (< 1%)	NS
Hyposaturation	4* (2%)	5**(2%)	NS
Oxygen therapy needed	19 (8%)	17 (7%)	NS
Hypotension	-	1 (< 1%)	NS
Nausea	5 (2%)	11 (5%)	NS
Vomiting	-	3 (1%)	NS
Antiemetics administration	13 (6%)	25 (11%) *#*	0.037

Data are presented as number and percentage; * Number of all the 7 reported cases of desaturation, 3 occurred prior to sufentanil administration and ** number of the 8 reported cases of desaturation, 3 occurred prior to sufentanil administration due to a chronic condition (chronic obstructive pulmonary disease). Antiemetics administered included ondansetron or thiethylperazine. *NS* not significant; # Statistically significant with presented *p*-value.

**Table 4:** Impact of intravenous sufentanil administration on changes in physiological parameters in trauma patients

Difference from initial physiologic values throughout to handover	Consultation (*n* = 233)	Competence (*n* = 229)	*p*-value
Systolic BP (mmHg)	−5.3 ± 15.6	−5,4 ± 12.8	NS
Diastolic BP (mmHg)	+ 1.8 ± 8.2	−2.5 ± 9.6 #	< 0.001
HR difference (bpm)^1^	−2.4 ± 10.7	−2.1 ± 9.5	NS
SpO2 (%)	+ 0.3 ± 3.5	−0.1 ± 2.2	NS
RR (breaths per minute)	−0.7 ± 2.1	−0.6 ± 1.7	NS
Glasgow Coma Scale	0 ± 0.3	0 ± 0.1	NS

Data are presented as mean ± standard deviation

* Abbreviations: BP* blood pressure; *HR* heart rate;* SpO₂* peripheral oxygen saturation; *RR* respiratory rate

Differences reflect changes between initial values and those recorded at handover in the emergency department. Negative sign means decrease from on-scene initial values. *NS* not significant; # Statistically significant with presented *p*-value

## Discussion

This prospective cohort study evaluated the effectiveness and safety of protocolized intravenous sufentanil administration by paramedics in the Czech Republic, comparing those authorized to administer independently after voluntary training with those requiring remote telephone consultation with an EMS physician. Our findings demonstrate that trained paramedics can safely and effectively manage moderate traumatic pain without physician supervision, supporting the potential expansion of paramedic competencies within the Czech PEC system.

Scene times did not differ significantly between groups, suggesting that the procedural delay introduced by physician phone consultation is minimal. However, paramedics in the Competency group administered a slightly but significantly higher dose of sufentanil compared to the Consultation group. This modest difference may reflect greater confidence in titrating opioids among independently authorized paramedics, aligning with international findings that autonomy improves pain management outcomes [3, 11, 12]. 

Moreover, the Competency group more frequently used adjuvant non-opioid analgesia, despite identical protocol permissions across groups. This suggests that voluntary, protocol-based training not only enhances opioid titration but also promotes proactive multimodal pain strategies, consistent with best practice in prehospital care [13]. 

Pain relief, measured via changes in the NRS score, was similar in both groups. However, complete documentation of pre- and post-administration pain scores was significantly more frequent in the Competency group, indicating better adherence to outcome monitoring protocols. This reflects improved clinical accountability and record quality when paramedics are specifically trained for autonomous opioid administration.

Regarding safety, adverse events such as respiratory depression, oxygen desaturation, hypotension, and nausea were rare and occurred at comparable rates between groups. Notably, the use of intravenous antiemetics was higher in the Competency group, despite similar rates of nausea and vomiting. This may reflect a more anticipatory and patient-centered approach by trained paramedics.

Only diastolic blood pressure showed a small but statistically significant decrease in the Competency group before patient handover, remaining within clinically acceptable ranges. Other vital signs, including heart rate, respiratory rate, SpO₂, and GCS, remained stable, confirming the hemodynamic safety of protocolized sufentanil use.

Our findings are consistent with previous studies from Germany and other European systems, which have shown that opioid analgesia administered by trained paramedics under strict protocols is both feasible and safe [3, 11, 13, 14]. Additionally, Le Cornec et al. compared ketamine to morphine for out-of-hospital analgesia and found similar efficacy between the two agents, but differing side effect profiles [15]. Ketamine was associated with a higher incidence of dissociation and agitation, while morphine had more nausea [15]. Nevertheless, low-dose sufentanil administration in less severe trauma in our study produced very low rates of adverse effects, particularly those that could impair communication and cooperation in prehospital settings [4, 16]. 

Overall, this study suggests that sufentanil administration by paramedics, when supported by voluntary structured training and protocol-based authorization, is safe, effective, and feasible in the Czech PEC system. Furthermore, it underscores the broader principle that enhancing paramedic autonomy, backed by rigorous education, can improve the quality of analgesia without compromising patient safety.

### Limitations

However, certain limitations must be acknowledged. As with all observational studies, the possibility of unmeasured confounding cannot be fully excluded. The monocentric study was also restricted to a single region, which may limit generalizability. The non-randomized design and potential selection biases related to paramedic decision-making may also affect the interpretation of the results. Furthermore, the study focused exclusively on adult patients with lower severity trauma, so the findings may not apply to patients with more severe injuries or other clinical conditions. Additionally, incomplete pain score documentation in the Consultation group could introduce measurement bias. Future multicenter trials and registry-based surveillance are recommended to validate these findings across different EMS systems and to explore the role of alternative agents such as ketamine in paramedic-administered analgesia.

## Conclusion

Intravenous sufentanil administered by trained paramedics within a protocolized competency framework appears to be safe and effective for pain management in adult patients with lower severity trauma during the prehospital phase. Compared to administration following remote physician consultation, autonomous administration demonstrated similar safety profiles, increased use of multimodal analgesia, and improved documentation practices. These findings offer practical and evidence-based guidance for the potential expansion of paramedic scope of practice in the Czech Republic.

## Supplementary Information

Below is the link to the electronic supplementary material.

Supplementary Material 1 Detailed paramedics, baseline and efficacy, adverse event and physiologic values are mentioned in tables of supplemental digital content

## Data Availability

No datasets were generated or analysed during the current study.

## Declarations

### **Use of AI and AI-assisted technologies in the writing process**

During the preparation of this manuscript, the authors utilized ChatGPT (OpenAI) to assist in correcting English grammar and enhancing language clarity. All content was reviewed and edited by the authors to ensure accuracy and integrity. The authors take full responsibility for the content of the manuscript.
