# Supplementary material for: Efficacy and safety of intravenous sufentanil administration in low-severity acute trauma as a competence of paramedics: a follow-up observational study
Source: Eur J Trauma Emerg Surg. 2025 Sep 9;51(1):289. doi: 10.1007/s00068-025-02953-0 (PMC12420757; doi:10.1007/s00068-025-02953-0)
Supplement: Supplementary file 1 — Supplementary Material 1 Detailed paramedics, baseline and efficacy, adverse event and physiologic values are mentioned in tables of supplemental digital content [file 68_2025_2953_MOESM1_ESM.docx]

**Supplemental Digital Content**

Title: Efficacy and Safety of Intravenous Sufentanil Administration in Low-Severity Acute Trauma as A Competence of Paramedics: A Follow-Up Observational Study

***Characteristics of paramedics*** ***in the EMS of the Karlovy Vary Region in 2024***

- Ambulance crew paramedics delivering PEC

n = 112

- Paramedics who lost competency at the beginning of year

n = 3

- Number of courses completed by paramedics with competency from 2020 to 2024

1 n = 16 (29%)

2 n = 10 (18%)

3 n = 26 (46%)

4 (lectors) n = 4 (7%)

**Table S1. Characteristics of paramedics in 2024 by competency to administer intravenous sufentanil in adult acute trauma: a comparison of two groups**

|  | Without competence  (n = 56) | Competence to administer (n = 56) | p-value |
| --- | --- | --- | --- |
| Age (years) | 45.9 ± 10.9 | 44.0 ± 10.0 | .94 |
| Gender (man) | 14 (25%) | 26 (46%) | .018 |
| Higher professional education | 37 (66%) | 31 (55%) | .29 |
| Bachelor’s degree | 15 (27%) | 16 (29%) |  |
| Master’s or higher degree | 4 (7%) | 9 (16%) |  |
| Length of praxis (years) | 14.6 ± 9.3 | 13.9 ± 7.7 | .32 |

*Data are presented as mean* ± *standard deviation or as number and percentage.*

**Table S2. Other characteristics**

|  | CONSULTATION  (n = 233) | COMPETENCE  (n = 229) | p-value |
| --- | --- | --- | --- |
| Urgency by MDC (I-IV range) | 3.0 ± 0.2 | 3.0 ± 0.1 | .10 |
| Transport time (min) | 22.6 ± 11.5 | 21.7 ± 14.6) | .52 |
| Additional i.v. paracetamol use  dose (mg) | 79 (34%)  983 ± 78 | 124 (54%)  992 ± 85 | <.001  .47 |
| Other additional analgesics  diclofenac  ketamin  ketamin/propofol  metamizole | 9 (4%)  4 (2%)  1 (<1%)  3 (1%) | -  -  -  - |  |
| NRS, initial (points 0-10) | 7.6 ± 1.7 | 7.4 ± 1.8 | .33 |
| Reported (NRS) in cases | *n = 180* | *n = 216* | - |
| NRS at handover (points 0-10) | 3.8 ± 2.0 | 3.4 ± 1.6 | .029 |
| Reported (NRS) in cases | *n = 169* | *n = 212* | - |

*Data are presented as mean* ± *standard deviation or as number and percentage. Abbreviations: MDC, medical dispatching center, NRS, numeric rating scale.*

**Table S3. Impact of intravenous sufentanil administration on physiological parameters in trauma patients**

|  | CONSULTATION  (n = 233) | | COMPETENCE  (n = 229) | | p-value |
| --- | --- | --- | --- | --- | --- |
|  | Initial | Handover | Initial | Handover |  |
| Systolic BP (mmHg) | 144.5  ± 23.3 | 139.0  ± 22.1 | 144.4  ± 21.0 | 139.0  ± 18.9 | .98  .98 |
| Systolic BP difference (mmHg) |  | -5.3 ± 15.6 |  | -5,4 ± 12.8 | .93 |
| Diastolic BP (mmHg) | 77.3 ± 11.6 | 79.2 ± 13.0 | 80.6 ± 11.2 | 78.0 ± 10.8 | .002  .27 |
| Diastolic BP difference (mmHg) |  | +1.8 ± 8.2 |  | -2.5 ± 9.6 | <0.001 |
| HR (bpm) | 84.8 ± 15.7 | 82.5 ± 13.6 | 84.0 ± 12.5 | 81.9 ± 11.8 | .53  .63 |
| HR difference  (bpm)^1^ |  | -2.4 ± 10.7 |  | -2.1 ± 9.5 | .75 |
| SpO2 (%) | 96.6 ± 3.7 | 96.9 ± 2,1 | 96.9 ± 2.4 | 96.7 ± 1.8 | .34  .32 |
| SpO2 difference (%) |  | +0.3 ± 3.5 |  | -0.1 ± 2.2 | .096 |
| RR  (breaths per minute) | 16.0 ± 2.6 | 15.3 ± 1.9 | 15.6 ± 2.4 | 14.9 ± 1.8 | .14  .03 |
| RR difference  (breaths per minute) |  | -0.7 ± 2.1 |  | -0.6 ± 1.7 | .74 |
| Glasgow Coma Scale (points) | 15.0 ± 0.3 | 15.0 ± 0.2 | 15.0 ± 0.1 | 15.0 ± 0.2 | .12  .97 |
| Glasgow Coma Scale  (difference) |  | 0 ± 0.3 |  | 0 ± 0.1 | .17 |

*Data are presented as mean* ± *standard deviation. Abbreviations: BP – blood pressure; HR – heart rate; SpO₂ – peripheral oxygen saturation; RR – respiratory rate. Differences reflect changes between initial values and those recorded at handover in the emergency department.*

**Table S4. Other reported conditions and aspects**

|  | CONSULTATION  (n = 233) | COMPETENCE  (n = 229) | p |
| --- | --- | --- | --- |
| Ebrietas | 8 (3%) | 6 (3%) | .61 |
| Medication used before EMS arrival | methoxyfluranum (n = 1)  pervitin (n = 1)  paracetamol/guaifenesinum (n = 1)  tramadol (n =1) | ibuprofenum (n = 2)  tramadol/paracetamol (n = 1)  paracetamol (n = 1)  metamizole (n =1)  clonazepam (n = 1)  kratom (n = 1) | -  -  -  -  -  - |

*Data are presented as absolute numbers, with percentages provided for cases of ebriety.*
